# Maternal Tetanus Toxoid Vaccination in Benin: Evidence from the Demographic and Health Survey

**DOI:** 10.3390/vaccines11010077

**Published:** 2022-12-29

**Authors:** Daniel Amoak, Nancy Osei Kye, Florence Wullo Anfaara, Yujiro Sano, Roger Antabe

**Affiliations:** 1 Department of Geography and Environment, Western University, London, ON N6A 3K7, Canada; 2 Department of Gender, Sexuality, and Women’s Studies, Western University, London, ON N6A 3K7, Canada; 3 Department of Sociology, Nipissing University, North Bay, ON P1B 8L7, Canada; 4 Department of Health and Society, University of Toronto Scarborough, Toronto, ON M5S 1A1, Canada

**Keywords:** tetanus, maternal tetanus toxoid vaccination, Benin, demographic and health survey

## Abstract

Tetanus toxoid vaccination is critical for improving maternal and child health. Yet, the prevalence and correlates of maternal tetanus toxoid vaccination coverage remain largely underexplored in Benin where infant and child mortality rates are high. Using the 2017–18 Benin Demographic and Health Survey, we apply logistic regression analysis to address this void in the literature. We find that overall maternal vaccination coverage is 69%. A range of demographic, health care, and socioeconomic factors are associated with maternal tetanus toxoid vaccination coverage. Women aged 20–34 (OR = 0.84, *p* < 0.05) and 35–49 (OR = 0.63, *p* < 0.01) are less likely to receive tetanus toxoid vaccination in comparison to those aged 15–19. Health care factors are also significantly associated with maternal tetanus toxoid vaccination, indicating that women who deliver at home (OR = 0.20, *p* < 0.001) and visit antenatal care fewer than eight times (OR = 0.62, *p* < 0.001) are less likely to receive tetanus toxoid vaccination than their counterparts who deliver in a health facility and visit antenatal care eight times or more. We also find that women with secondary (OR = 0.54, *p* < 0.05), primary (OR = 0.47, *p* < 0.01), and no education (OR = 0.47, *p* < 0.01) are less likely to receive tetanus toxoid vaccination compared to their counterparts with higher education. Based on these findings, we discuss several implications for policymakers.

## 1. Introduction

According to the World Health Organization (WHO), tetanus toxoid (TT) infection remains one of the major contributors to maternal and child mortality in regions such as Sub-Saharan Africa (SSA). This preventable disease is transmitted through contaminated wounds and is often found in individuals who have not received the necessary vaccinations or booster shots to maintain immunity [1]. It is estimated that nearly all of the reported cases of tetanus are birth-associated in newborns and mothers who have not received adequate tetanus-toxoid vaccines (TTV) [1]. Emphasising on the importance of TTV for maternal and child health, it has been observed that, about 96% reduction in neonatal tetanus was achieved following intensive health promotion campaigns and disease control strategies implemented at the regional, national, and international levels [2].

In a global attempt to control and eliminate TT infection, the WHO in collaboration with the UN and UNICEF instituted The Maternal and Neonatal Tetanus Elimination (MNTE) Initiative in 1999 to identify and prioritize increasing TTV to 59 countries mostly in SSA with a high prevalence of TT infection. Furthermore, in both the millennium development goals (MDGs) and sustainable development goals (SDGs), the eradication of TT has been recommended through goals that aim at increasing vaccine coverage for mothers and newborns. While progress continues to be made, as of 2020, 12 countries have still not reached the MNTE status with dire consequences for maternal and child mortality. For instance, in 2015, about 34,000 neonatal deaths were caused by TT, the majority of whom were reported in SSA [1]. Particularly in areas with low immunization such as Benin, it is reported that there is a near 100% case-fatality rates among neonates [3,4].

Specifically, Benin is lacking widespread coverage of TTV, making it a challenge for reducing or achieving the MNTE [5]. It is further documented that the TTV coverage in the country may be worsening as it dropped from 81% in 2000 to 69% in 2018, that is short of the national objective of 86% [3,6]. These worsening TTV coverage dynamics may be contributing towards Benin’s high maternal and infant mortality rates. For example, Benin reports higher maternal mortality rate of 379 deaths per 100,000 live births, which is substantially higher than the global average of 211 deaths per 100,000 live births. A similar trend has been observed with infant mortality where Benin records 86 deaths per 1000 live births in comparison to the global average of 29 deaths per 1000 live births [7].

Reflecting on the low coverage and uptake of TTV among women in Benin and its associated impact on maternal and child mortality rates, studies suggest that there may be some barriers that need to be addressed to achieve MNTE. These studies point to a range of demographic, health care, and socioeconomic factors that may be influencing the uptake of TTV in Benin and elsewhere in SSA [2,8,9]. In Nigeria and Ethiopia, for example, socioeconomic conditions such as household wealth and level of maternal education were associated with TTV uptake among pregnant women [10,11]. The low uptake of TTV was also higher among women who lived further from a health facility [12]. In Sierra Leone and Somalia, studies also showed that mothers living in urban areas were more likely to receive the double vaccine than those in rural areas [12,13]. Other predictors include the number of antenatal care (ANC) visitations and place of delivery [13,14,15]. The impact of ANC visitation is particularly important as expectant mothers are not only informed about recommended lifestyle and dietary options to safeguard their health and that of their unborn babies, but they also have the opportunity to receive TTV. In Sudan, Mohamed and Ahmed [16] observed that the frequency of ANC visits contributed to the coverage of TTV among pregnant women.

Despite the relevance of these studies, there is little knowledge on the factors associated with TTV uptake during pregnancy in Benin. As one of the priority countries where maternal and neonatal TT infection poses a substantial public health challenge, it is critical to understand the factors that may be driving the uptake of TTV among women [3]. To this end, we use a nationally representative survey to explore the prevalence and correlates of the uptake of TTV among pregnant women in Benin.

## 2. Methods

### 2.1. Data

We used the 2017–18 Benin Demographic and Health Survey (BDHS), which was collected by the National Institute of Statistics and Economic Analysis under the supervision of the Ministry of Planning and Development, with technical assistance with ICF International through the USAID-funded DHS program. The BDHS is suitable for this study with high-quality and reliable information on basic demographic indices and health indicators including the uptake of TTV. The BDHS employed a two-stage sampling framework, in which a stratified probability proportional to size sampling method was applied and conducted face-to-face interviews with 15,928 women and 7595 men with a response rate of 98% for both women and men. For the purpose of this study, we focused on 8412 women who have given birth in the last five years and answered questions about TTV.

### 2.2. Measures

Based on the WHO recommendation [3], we create the dependent variable called ‘maternal TTV’ where women are coded as ‘yes’ when they receive (1) at least two TT injections during the birth, (2) two or more injections within three years of the birth, (3) three or more injections within five years of the birth, (4) four or more injection within 10 years of the birth, or (5) five or more injections at any time prior to the birth (0 = no; 1 = yes). Informed by previous research [13,17], we also introduce three sets of independent variables, namely demographic, health care, and socioeconomic factors. Demographic factors include age of respondents (0 = 15–19; 1 = 20–34; 2 = 35–49), birth order (0 = first; 1 = second; 2 = third; 3 = fourth; 4 = fifth+), marital status (0 = formerly married; 1 = currently married; 2 = never married), religion (0 = Christian; 1 = traditionalist; 2 = Muslim; 3 = no religion/other), and place of residence (0 = urban; 1 = rural). We also include two factors related to health care, namely place of delivery (0 = health facility; 1 = home) and number of ANC visits (0 = eight times or more; 1 = fewer than eight times). Finally, there are two socioeconomic variables such as education (0 = higher education; 1 = secondary education; 2 = primary education; 3 = no education) and household wealth quintile (0 = richest; 1 = richer; 2 = middle; 3 = poorer; 4 = poorest).

### 2.3. Data Analysis

We employ three different analyses for this study. First, we use univariate analysis to describe the characteristics of our analytical sample. Second, bivariate analysis is conducted to understand the unadjusted associations between the dependent and independent variables. Third, we rely on multivariate analysis to simultaneously include the independent variables to produce adjusted estimates. For bivariate and multivariate analysis, we use logistic regression analysis due to the dichotomous nature of the dependent variable [18]. Results are shown with odds ratios (ORs). ORs larger than 1 indicate that women are more likely to receive TTV while those smaller than 1 point to lower odds of receiving it. All analyses are carried out using STATA 17 (State Corp, College Station, TX, USA). The ‘svy’ function is applied in statistical analysis to adjust for the cluster sampling design as well as sampling weights.

## 3. Results

Table 1 shows sample characteristics. We find that 69% of women received TTV. The largest age group is 20–34 (70%) followed by 15–19 (24%) and 35–49 (6%). The majority of women were currently married (93%). While more women live in rural areas (39%) than rural areas (61%), the largest religious group is Christian (50%) followed by Muslim (34%) and traditionalist (9%). It is also important that the majority deliver in a health facility (86%); however, less than one in ten women visit ANC eight times or more (9%). In terms of socioeconomic status, we find that the majority of women do not have any formal education (64%).

Table 2 shows bivariate and multivariate findings. At the bivariate level, we find that a range of demographic, health care, and socioeconomic factors are significantly associated with maternal TTV. For example, women aged 20–34 years (OR = 0.84, *p* < 0.01) and 35–49 years (OR = 0.55, *p* < 0.001) are less likely to receive TTV in comparison to those aged 15–19. In addition, we find that Muslim women (OR = 0.47, *p* < 0.001) and women with other religion or no religion (OR = 0.55, *p* < 0.001) are both less likely to receive TTV compared to their Christian counterparts. Odds of receiving TTV are also lower for women in rural areas in comparison to those in urban areas (OR = 0.59, *p* < 0.001). Health care factors are also significantly associated with maternal TTV, indicating that women who deliver at home (OR = 0.14, *p* < 0.001) and visit ANC fewer than eight times (OR = 0.36, *p* < 0.001) are less likely to receive TTV than their counterparts who deliver in a health facility and visit ANC eight times or more. For socioeconomic status, we find that women with secondary (OR = 0.36, *p* < 0.001), primary (OR = 0.28, *p* < 0.001), and no education (OR = 0.19, *p* < 0.001) are less likely to receive TTV compared to their counterparts with higher education. Similarly, compared to those who belong to the richest category, women who belong to the ‘richer’ (OR = 0.71, *p* < 0.001), ‘middle’ (OR = 0.45, *p* < 0.001), ‘poorer’ (OR = 0.36, *p* < 0.001), and ‘poorest’ (OR = 0.20, *p* < 0.001) category are less likely to receive TTV.

Multivariate results are largely consistent with bivariate results. For example, women aged 20–34 years (OR = 0.84, *p* < 0.05) and 35–49 years old (OR = 0.63, *p* < 0.01) are less likely to receive TTV in comparison to those aged 15–19 years. We also find that Muslim women (OR = 0.74, *p* < 0.001) are less likely to receive TTV compared to their Christian counterparts. Odds of receiving TTV are also lower for women in rural areas in comparison to those in urban areas (OR = 0.84, *p* < 0.05). Health care factors are also significantly associated with maternal TTV, indicating that women who deliver at home (OR = 0.20, *p* < 0.001) and visit ANC fewer than eight times (OR = 0.62, *p* < 0.001) are both less likely to receive TTV than their counterparts who deliver in a health facility and visit ANC eight times or more. For socioeconomic status, we find that women with secondary (OR = 0.54, *p* < 0.05), primary (OR = 0.47, *p* < 0.01), and no education (OR = 0.47, *p* < 0.01) are less likely to receive TTV compared to their counterparts with higher education. Similarly, compared to those who belong to the richest category, women who belong to the ‘middle’ (OR = 0.68, *p* < 0.001), ‘poorer’ (OR = 0.62, *p* < 0.001), and ‘poorest’ (OR = 0.43, *p* < 0.001) category are less likely to receive TTV.

## 4. Discussion

In line with the SDG3 to reduce maternal and child mortality, there has been an increased emphasis on TTV for women, particularly in contexts such as Benin with high rates of maternal and child mortality. As part of its overall strategies to reduce maternal and child mortality, the Government of Benin has been working to increase TTV among especially pregnant women in addition to other critical vaccines such as diphtheria and polio [19,20]. While these policy initiatives are important, there is still dearth of studies in Benin examining the prevalence and correlates of TTV in the country. To fill this gap and to inform policy on maternal and child health, our study examined the prevalence and determinants of TTV among women in Benin.

Overall, our findings reveal that a significant number of women in Benin are not receiving TTV. Specifically, only 69% of women in the country reported having been vaccinated against TT. This is far below the WHO-recommended 100% TTV coverage for women in the settings of high maternal and child mortality like Benin. The low coverage of TTV among expectant mothers has also been reported by earlier studies such as Togora and colleagues [20], who observed in the Health Zone Zogbodomey–Bohicon–Zakpota of Benin that only 62% of pregnant women received TTV. These revelations call for more efforts to increase the coverage of TTV among women, especially as the country strives to meet WHO’s maternal and child mortality targets by 2030.

We also found a range of demographic and socioeconomic factors to be associated with TTV among women in Benin. For instance, the oldest age category of women was less likely to have received TTV compared to the youngest age category that is 15–19 years. This finding may contrast with that of earlier studies such as Belay et al. [21] and Yaya et al.’s [13] finding that older women in East Africa and Sierra Leone were more likely to receive TTV due to their exposure to information on TT. It is possible that younger women may be experiencing their first births, which may lead to higher risk perceptions associated with their pregnancy. They may therefore be more inclined to receive vaccinations to protect their health and that of their unborn babies. Supporting this argument, Edgard-Marius et al. [22] also reported in South Benin that younger expectant mothers out of fear and anxiety for pregnancy-related complications were better positioned to attend antenatal care visits relative to their older counterparts.

In addition, we also found that religious affiliations were associated with TTV coverage among women. Compared to their Christian colleagues, Muslim women were found to be less likely to have received TTV. This finding is consistent with earlier findings in Nigeria where it was observed that expectant mothers who were Muslim had the lowest motivation towards vaccinating their unborn children relative to their Christian counterparts [23]. Reflecting on these findings, Adeyanju et al. [23] and Anyene [24] opine that heightened vaccine hesitancy among Muslim women may be hinged on issues of trust in vaccines which is further demonstrated in the low uptake of vaccination in a similar religious context. In addressing this challenge, it is critical for public health practitioners to work to increase women’s trust in vaccines through their community and religious leadership.

We also observed that women in rural areas of Benin were less likely to report having TTV compared to their urban counterparts. This finding is largely consistent with earlier studies that have alluded to an urban advantage in vaccination against TT in varying areas in SSA [12,17]. Specifically, urban women in Benin and the rest of SSA enjoy improved access to health information and health care facilities and personnel. This urban bias in access to health care resources may work to increase their chances of receiving the range of vaccinations including TT before and during pregnancy and after birth relative to their rural counterparts where such health care facilities and personnel are mostly lacking [25,26,27].

Our study further revealed that health care factors were also associated with the coverage of TTV among women in Benin. Expectant mothers who delivered at home or did not meet all the eight recommended number of ANC visits were less likely to have been vaccinated against TT. This finding is consistent with previous research, indicating that women who have frequent interactions with health care personnel by meeting the recommended ANC visits may have an increased awareness of the benefits of TTV [17]. During these ANC visits, expectant mothers receive health education on lifestyle and dietary choices, as well as information on preventive health care practices including the usefulness of TTV in protecting the mother and unborn child [28,29]. Given the health promotion activities at health care facilities targeted at expectant mothers, several studies have revealed similar findings where women with frequent ANC visits and delivering in recommended health care facilities were more likely to report receiving TTV relative to their counterparts who did not meet the total number of ANC visits or delivered at home [21,30].

A range of socioeconomic factors were also found to be associated with the uptake of TTV among women in Benin. Women with secondary, primary, or no formal education were found to be less likely to have been vaccinated against TT compared to their colleagues who have attained tertiary education. This finding is consistent with that of existing studies in Benin and elsewhere in SSA, suggesting that women with higher educational attainment tend to be more proactive in meeting their health care needs and that of their unborn babies and a such, are more likely to meet the recommended number of ANC visits [10,21,31,32]. It has been argued that higher educational attainment increases women’s self-awareness about their health care needs and they are more likely to take up preventive health behaviours that are intended to safeguard their health and that of their families. In the context of our findings and consistent with the observation by Belay et al. [21] in Ethiopia, it is possible that expectant mothers with tertiary levels of education fully appreciate the benefits associated with the uptake of TTV in protecting themselves and their unborn children. Consistent with the literature, we also found that women from the richest households were more likely to have received TTV during pregnancy relative to those from the richer, middle, poor and poorest households. In unpacking these findings, Liyew and Ayalew [17] explain in Ethiopia that household wealth serves as an important determinant of women’s utilization of health services including vaccinations as it removes the financial barriers that may be associated with the utilization of maternal health services. Specifically, Budu and colleagues [30] also found in Benin that household wealth played a critical role in women’s immunization of their children aged 12–23 months.

There are some noteworthy limitations associated with our study. First the BDHS is a cross-sectional survey, suggesting that our findings are limited to statistical association and must therefore be interpreted with caution. Second, our responses may be subject to recall bias given that study participants were asked to recall if they had received TTV before, during and after periods of pregnancy. Indeed, this bias may be relevant in this study, considering that the BDHS had to rely on the mother’s verbal report to confirm TTV status when they did not have vaccination cards. Finally, the decision to receive TTV may be impacted by other contextual factors including the availability of the vaccine in health care facilities, distance to health facilities, as well as perceptions about the efficacy of the vaccine. Future studies may want to explore all these variables that may impact the coverage of TTV among women before, during and after pregnancy. Despite these limitations, our study is among the first to examine the national-level prevalence and correlates of TTV coverage among women in Benin.

## 5. Conclusions

Based on our study findings, we have some suggestions for policymakers. For Benin to make progress in achieving SDG3, there is an urgent need to increase the coverage of TTV from the low of 69% to the UN-recommended level of 100%. Increasing TTV coverage among women may be essential for achieving a 94% reduction in all neonatal maternal deaths related to TT infection [33]. In addition, it may also be critical to closely collaborate with religious leaders to increase women’s uptake of TTV through community-based sensitization campaigns on the importance, safety and efficacy of TTV. Given the observed benefits of women’s access to ANC and delivering at a recommended health facility on the uptake of TTV, it may be useful for campaign programs in Benin to promote the use of ANC in the context of TTV uptake. Finally, we found socioeconomic disparities in the uptake of TTV, which may be pointing to the need of addressing structural barriers to TTV coverage.

## Figures and Tables

**Table 1:** Sample characteristics.

Variable	Percentage
*Maternal TT vaccination*	
No	31
Yes	69
*Age of respondents*	
15–19	24
20–34	70
35–49	6
*Birth order*	
First	20
Second	18
Third	17
Fourth	14
Fifth+	31
*Marital status*	
Formerly married	4
Currently married	93
Never married	3
*Religion*	
Christian	50
Traditionalist	9
Muslim	34
No religion/other	7
*Place of residence*	
Urban	39
Rural	61
*Place of delivery*	
Health facility	86
Home	14
*Number of ANC visit*	
Eight time or more	9
Fewer than eight times	91
*Education*	
Higher education	2
Secondary education	16
Primary education	18
No education	64
*Household wealth quintile*	
Richest	19
Richer	21
Middle	21
Poorer	20
Poorest	19
Total	8412

**Table 2:** Unadjusted and adjusted logit models predicting ‘maternal TT vaccination’ in Benin.

	Unadjusted			Adjusted		
	OR	95% CI		OR	95% CI	
*Age of respondents*						
15–19	1.00			1.00		
20–34	0.84 **	0.75	0.94	0.84 *	0.72	0.98
35–49	0.55 ***	0.44	0.70	0.63 **	0.47	0.85
*Birth order*						
First	1.00			1.00		
Second	0.99	0.85	1.15	0.98	0.81	1.17
Third	1.10	0.93	1.29	1.14	0.94	1.39
Fourth	1.12	0.95	1.32	1.20	0.98	1.46
Fifth+	0.96	0.83	1.12	1.18	0.96	1.45
*Marital status*						
Formerly married	1.00			1.00		
Currently married	0.90	0.69	1.16	0.99	0.76	1.29
Never married	0.88	0.62	1.25	0.89	0.60	1.32
*Religion*						
Christian	1.00			1.00		
Traditionalist	0.87	0.70	1.08	1.22	0.98	1.51
Muslim	0.47 ***	0.39	0.55	0.74 ***	0.64	0.87
No religion/other	0.55 ***	0.43	0.71	0.97	0.74	1.26
*Place of residence*						
Urban	1.00			1.00		
Rural	0.59 ***	0.50	0.70	0.84 *	0.72	0.98
*Place of delivery*						
Health facility	1.00			1.00		
Home	0.14 ***	0.11	0.17	0.20 ***	0.17	0.25
*Number of ANC visit*						
Eight time or more	1.00			1.00		
Fewer than eight times	0.36 ***	0.28	0.45	0.62 ***	0.48	0.80
*Education*						
Higher education	1.00			1.00		
Secondary education	0.36 ***	0.21	0.63	0.54 *	0.30	0.95
Primary education	0.28 ***	0.16	0.48	0.47 **	0.26	0.83
No education	0.19 ***	0.11	0.32	0.47 **	0.26	0.82
*Household wealth quintile*						
Richest	1.00			1.00		
Richer	0.71 ***	0.57	0.87	0.89	0.72	1.11
Middle	0.45 ***	0.36	0.55	0.68 ***	0.54	0.85
Poorer	0.36 ***	0.29	0.45	0.62 ***	0.49	0.80
Poorest	0.20 ***	0.16	0.25	0.43 ***	0.34	0.55

* *p* < 0.05, ** *p* < 0.01, *** *p* < 0.001; OR = odds ratio; CI = confidence interval.

## Data Availability

Available at https://dhsprogram.com/data (accessed on 11 September 2022).

## References

[1] World Health Organization Tetanus: Key Facts. https://www.who.int/news-room/fact-sheets/detail/tetanus.

[2] World Health Organization (2019). Protecting All against Tetanus: Guide to Sustaining Maternal and Neonatal Tetanus Elimination (MNTE) and Broadening Tetanus Protection for All Populations.

[3] Njuguna H.N., Yusuf N., Raza A.A., Ahmed B., Tohme R.A., Nations F. (2020). Weekly Epidemiological Record Relevé Épidémiologique Hebdomadaire. Wkly. Epidemiol. Rec..

[4] mondiale de la Santé O. (2020). World Health Organization Maternal and Neonatal Tetanus Eliminated in the South-West Geopolitical Zone of Nigeria. Wkly. Epidemiol. Rec..

[5] Kanu F.A., Yusuf N., Kassogue M., Ahmed B., Tohme R.A. (2022). Progress Toward Achieving and Sustaining Maternal and Neonatal Tetanus Elimination—Worldwide, 2000–2020. MMWR Recomm. Rep..

[6] Togora M., Kpozèhouen A., Saizonou J., Sossa C., Ouégraogo L., Makoutodé M. (2014). Facteurs Associes a La Faible Couverture En Vaccin Antitetanique Chez Les Femmes Enceintes Dans La Zone Sanitaire de Zogbodomey-Bohicon-Zakpota Au Benin. Mali Med..

[7] UNICEF Country Profile: Benin Key Demographic Indicators. https://data.unicef.org/country/ben/.

[8] Faria A.P.V., da Silva T.P.R., Duarte C.K., Mendes L.L., Santos F.B.O., Matozinhos F.P. (2021). Tetanus Vaccination in Pregnant Women: A Systematic Review and Meta-Analysis of the Global Literature. Public Health.

[9] Yusuf N., Raza A.A., Chang-Blanc D., Ahmed B., Hailegebriel T., Luce R.R., Tanifum P., Masresha B., Faton M., Omer M.D. (2021). Progress and Barriers towards Maternal and Neonatal Tetanus Elimination in the Remaining 12 Countries: A Systematic Review. Lancet Glob. Health.

[10] Mamoro M.D., Hanfore L.K. (2018). Tetanus Toxoid Immunization Status and Associated Factors among Mothers in Damboya Woreda, Kembata Tembaro Zone, SNNP, Ethiopia. J. Nutr. Metab..

[11] Ogundare E.O., Ajite A.B., Adeniyi A.T., Babatola A.O., Taiwo A.B., Fatunla O.A., Airemionkhale A., Odeyemi O.A., Olatunya O.S., Oyelami O.A. (2021). A Ten-Year Review of Neonatal Tetanus Cases Managed at a Tertiary Health Facility in a Resource Poor Setting: The Trend, Management Challenges and Outcome. PLoS Negl. Trop. Dis..

[12] Gebremedhin T.S., Welay F.T., Mengesha M.B., Assefa N.E., Werid W.M. (2020). Tetanus Toxoid Vaccination Uptake and Associated Factors among Mothers Who Gave Birth in the Last 12 Months in Errer District, Somali Regional State, Eastern Ethiopia. Biomed. Res. Int..

[13] Yaya S., Kota K., Buh A., Bishwajit G. (2020). Prevalence and Predictors of Taking Tetanus Toxoid Vaccine in Pregnancy: A Cross-Sectional Study of 8,722 Women in Sierra Leone. BMC Public Health.

[14] Barrow A., Barrow S., Jobe A. (2022). Differentials in Prevalence and Correlates on Uptake of Tetanus Toxoid and Intermittent Preventive Treatment with Sulfadoxine-Pyrimethamine during Pregnancy: A Community-Based Cross-Sectional Study in The Gambia. SAGE Open Med..

[15] Iqbal S., Ali I., Ekmekcioglu C., Kundi M. (2020). Increasing Frequency of Antenatal Care Visits May Improve Tetanus Toxoid Vaccination Coverage in Pregnant Women in Pakistan. Hum. Vaccines Immunother..

[16] Mohamed S.O.O., Ahmed E.M. (2022). Prevalence and Determinants of Antenatal Tetanus Vaccination in Sudan: A Cross-Sectional Analysis of the Multiple Indicator Cluster Survey. Trop. Med. Health.

[17] Liyew A.M., Ayalew H.G. (2021). Individual and Community-Level Determinants of Poor Tetanus Toxoid Immunization among Pregnant Women in Ethiopia Using Data from 2016 Ethiopian Demographic and Health Survey; Multilevel Analysis. Arch. Public Health.

[18] Menard S. (2002). Applied Logistic Regression Analysis.

[19] WHO in Africa Benin Organizes the First States General of Vaccination. https://www.afro.who.int/fr/news/le-benin-organise-les-premiers-etats-generaux-de-la-vaccination.

[20] Togora M., Kpozehouen A., Saizonou J., Sossa C., Quegraogo L., Makoutodé M. (2014). Factors Associated with Low Coverage of Tetanus-Toxoid Vaccine in Pregnant Women in the Health Zone Zogbodomey- Bohicon-Zakpota, Benin. Mali Med..

[21] Belay A.T., Fenta S.M., Agegn S.B., Muluneh M.W. (2022). Prevalence and Risk Factors Associated with Rural Women’s Protected against Tetanus in East Africa: Evidence from Demographic and Health Surveys of Ten East African Countries. PLoS ONE.

[22] Edgard-Marius O., Charles S.J., Jacques S., Justine G.C.-C., Virginie M.A., Ibrahim M.A., Laurent O.T. (2015). Determinants of Low Antenatal Care Services Utilization during the First Trimester of Pregnancy in Southern Benin Rural Setting. Univers. J. Public Health.

[23] Adeyanju G.C., Sprengholz P., Betsch C. (2022). Understanding Drivers of Vaccine Hesitancy among Pregnant Women in Nigeria: A Longitudinal Study. NPJ Vaccines.

[24] Anyene B.C. (2014). Routine Immunization in Nigeria: The Role of Politics, Religion and Cultural Practices. Afr. J. Health Econ..

[25] Atuoye K.N., Dixon J., Rishworth A., Galaa S.Z., Boamah S.A., Luginaah I. (2015). Can She Make It? Transportation Barriers to Accessing Maternal and Child Health Care Services in Rural Ghana. BMC Health Serv. Res..

[26] Gessesse D.N., Yismaw A.E., Yismaw Y.E., Workneh T.W. (2021). Coverage and Determinants of Protective Dose Tetanus Toxoid Vaccine among Postnatal Women Delivered at University of Gondar Comprehensive Specialized Hospital, Northwest Ethiopia, 2019. Clin. Epidemiol. Glob. Health.

[27] Antabe R., Kansanga M., Sano Y., Kyeremeh E., Galaa Y. (2020). Utilization of Breast Cancer Screening in Kenya: What Are the Determinants ?. BMC Health Serv. Res..

[28] Atuoye K.N., Amoyaw J.A., Kuuire V.Z., Kangmennaang J., Boamah S.A., Vercillo S., Antabe R., McMorris M., Luginaah I. (2017). Utilisation of Skilled Birth Attendants over Time in Nigeria and Malawi. Glob. Public Health.

[29] Kuuire V.Z., Kangmennaang J., Atuoye K.N., Antabe R., Boamah S.A., Vercillo S., Amoyaw J.A., Luginaah I. (2017). Timing and Utilisation of Antenatal Care Service in Nigeria and Malawi. Glob. Public Health.

[30] Budu E., Seidu A.A., Agbaglo E., Armah-Ansah E.K., Dickson K.S., Hormenu T., Hagan J.E., Adu C., Ahinkorah B.O. (2021). Maternal Healthcare Utilization and Full Immunization Coverage among 12–23 Months Children in Benin: A Cross Sectional Study Using Population-Based Data. Arch. Public Health.

[31] Raru T.B., Ayana G.M., Zakaria H.F., Merga B.T. (2022). Association of Higher Educational Attainment on Antenatal Care Utilization Among Pregnant Women in East Africa Using Demographic and Health Surveys (DHS) from 2010 to 2018: A Multilevel Analysis. Int. J. Womens. Health.

[32] Muyunda B., Makasa M., Jacobs C., Musonda P., Michelo C. (2016). Higher Educational Attainment Associated with Optimal Antenatal Care Visits among Childbearing Women in Zambia. Front. Public Health.

[33] Blencowe H., Lawn J., Vandelaer J., Roper M., Cousens S. (2010). Tetanus Toxoid Immunization to Reduce Mortality from Neonatal Tetanus. Int. J. Epidemiol..

